# Anti‐inflammatory potential of remimazolam: A laboratory and clinical investigation

**DOI:** 10.1002/iid3.1218

**Published:** 2024-03-14

**Authors:** Shota Tsukimoto, Atsuhiro Kitaura, Hidetaka Kuroda, Uno Imaizumi, Fumihiko Yoshino, Ayaka Yoshida, Shinchi Nakao, Noriyuki Ohta, Yasuhumi Nakajima, Takuro Sanuki

**Affiliations:** ^1^ Department of Dental Anesthesiology Kanagawa Dental University Yokosuka Kanagawa Japan; ^2^ Department of Anesthesiology Kindai University Faculty of Medicine Osakasayama Osaka Japan; ^3^ Department of Pharmacology Kanagawa Dental University Yokosuka Kanagawa Japan; ^4^ Department of Dental Education Kanagawa Dental University Yokosuka Kanagawa Japan; ^5^ Perioperative Management Center Okanami General Hospital Iga Mie Japan

**Keywords:** anti‐inflammatory effect, antioxidant effect, C‐reactive protein (CRP), dexmedetomidine (Precedex®), electron spin resonance (ESR), remimazolam (Anerem®)

## Abstract

**Background:**

Anesthetic agents, particularly intravenous anesthetics, may affect immune function and tumorigenic factors. We herein investigated whether the anti‐inflammatory effects of anesthetic agents are attributed to their antioxidant properties. The antioxidant and anti‐inflammatory effects of remimazolam, a new anesthetic, remain unclear. We hypothesized that remimazolam exerts anti‐inflammatory effects due to its antioxidant properties, which may affect the postoperative inflammatory response. This retrospective clinical study examined this hypothesis using laboratory and clinical approaches.

**Methods:**

The antioxidant effects of remimazolam and dexmedetomidine were assessed by electron spin resonance (ESR) spectroscopy, and postoperative inflammatory responses were compared in 143 patients who underwent transcatheter aortic valve replacement at Kindai University Hospital between April 2021 and December 2022. The primary endpoint was the presence or absence of the antioxidant effects of the anesthetics themselves using ESR.

**Results:**

Remimazolam at clinical concentrations exerted antioxidant effects, whereas dexmedetomidine did not. Increases in C‐reactive protein (CRP) levels on POD3 from preoperative values were significantly smaller in the remimazolam group than in the dexmedetomidine group (1.33 ± 1.29 vs. 2.17 ± 1.84, *p* = .014).

**Conclusions:**

Remimazolam exerted stronger anti‐inflammatory effects than dexmedetomidine, and these effects were enhanced by its antioxidant properties, which may have affected postoperative CRP production.

## Graphical abstract

Remimazolam at clinical use concentrations has the power to scavenge hydroxyl radicals in a concentration‐dependent manner.

## 1 INTRODUCTION

Remimazolam, a new intravenous anesthetic, selectively binds to benzodiazepine receptors with high affinity and has a short duration of action. It is expected to improve the quality of anesthesia because it (1) does not cause vascular pain during its intravenous administration, (2) has stable circulatory dynamics, (3) has competitive antagonists, (4) is hydrolyzed by tissue esterases, and (5) has a shorter half‐life than other benzodiazepines.^1^, ^2^, ^3^, ^4^, ^5^ We developed our own anesthesia protocol for transcatheter aortic valve replacement (TAVR) that uses remimazolam or dexmedetomidine to maintain spontaneous breathing. The total length of the hospital stay and the total amount of circulatory agonists used are expected to be lower with remimazolam or dexmedetomidine than with general anesthesia.^6^

Anesthetic agents have the potential to affect immune function and tumorigenic factors. Specifically, intravenous anesthetics have been reported to reduce the levels of acute inflammatory cytokines (e.g., tumor necrosis factor‐α and interleukin 6 [IL‐6]) produced as a result of invasive surgical procedures.^7^, ^8^, ^9^ Previous studies compared various anesthetic agents and their impact on survival and recurrence rates after cancer surgery.^10^, ^11^, ^12^, ^13^, ^14^

Dexmedetomidine acts on (α‐) 2‐adrenergic receptors and exerts its anti‐inflammatory effects through changes in inflammatory cytokine production via its downstream signaling pathways.^15^, ^16^ On the other hand, midazolam, a benzodiazepine receptor agonist, acts on peripheral benzodiazepine receptors and contributes to the inhibition of acute inflammatory cytokine production via the NF‐kb pathway.^17^ Remimazolam also acts on peripheral benzodiazepine receptors.^18^ Although receptor‐mediated anti‐inflammatory effects have been reported, both drugs have only been tested in vitro.

A close relationship exists between oxidant stress and inflammation. The hydroxyl radical (OH^•^) is one of the reactive oxygen species (ROS) that are closely involved in inflammation.^19^, ^20^ In the present study, we focused on the anti‐inflammatory effects of anesthetics themselves due to their antioxidant properties. The anti‐inflammatory and antioxidant effects of remimazolam, a new benzodiazepine anesthetic drug, currently remain unclear, and few studies have examined its involvement in the immune system.

We hypothesized that remimazolam exerts anti‐inflammatory effects due to its antioxidant properties, which may affect the postoperative inflammatory response. To test this hypothesis, we employed both laboratory and clinical approaches. The ROS‐scavenging activities of the anesthetics were investigated by electron spin resonance (ESR) spectroscopy. We also investigated whether the ROS‐scavenging activities of the anesthetics affected postoperative inflammatory findings in TAVR patients using blood sampling.

## 2 MATERIALS AND METHODS

### 2.1 Laboratory study section: Evaluation of ROS‐scavenging activities with ESR

The JES‐RE 1X, X‐band spectrometer (JEOL) was used under a microwave power of 8.00 mW, magnetic field of 335.8 ± 7.5 mT, field modulation width of 0.079 mT, sweep time of 1 min, and time constant of 0.03 s. Measurement data were acquired using the software WIN RAD ESR Data Analyzer ver. 1.30 (Radical Research Inc.) with a gain of 320 and 4096 data points were recorded. All experiments were performed a minimum of four times.

The ESR technique, which specifically measures ROS, was used to measure OH^•^ generated by the hydrogen peroxide (H_2_O_2_, 100 mM, FUJIFILM Wako Pure Chemical Industries, Ltd.) + ultraviolet (UV, emission: 365 nm, 10 mW, 10 s, SUPERCURE‐204S, SAN‐EI ELECTRIC Co., Ltd.) system.^21^ Distilled water was used as the negative control. The spin trapping agent used was 5 mM 5‐(5,5‐dimethyl‐2‐oxo‐1,3,2‐dioxaphosphorinan‐2‐yl)−5‐methyl‐1‐pyrroline‐1‐oxide (CYPMPO, Mikuni Pharmaceutical Industrial Co., Ltd.). Anerem® (remimazolam besilate, Mundiphama K. K.) and Precedex® (dexmedetomidine hydrochloride, Pfizer Japan Inc.) were added to these products to assess their ROS‐scavenging activities.

OH^•^ was produced by H_2_O_2_ under UV irradiation. The intensity of the CYPMPO‐OH^•^ spin adduct was measured at varying concentrations of anesthetic drugs. The intensity of the spectrum was evaluated using a previously reported method.^21^ Spectral intensity following the addition of distilled water was set as I_0_, which was the baseline. The spectral intensity of each anesthetic was set as I. The ratio of I to I_0_ was used to evaluate the relative spectrum intensity (%Intensity of I/I_0_). This was assessed by a nonlinear regression of experimental data using the Lorentz function with OriginPro 2020 software ver. 9.7.0.188 (OriginLab Corp.). Formulations containing additives and other ingredients were used in this study.

### 2.2 Clinical study section: Study population and protocol of anesthesia

This retrospective observational study was conducted with the approval by the Research Ethics Board of Kindai University Faculty of Medicine (approval no. R03‐123) and according to the principles of the Declaration of Helsinki. It was registered before patient enrollment at https://center6.umin.ac.jp/cgi-open-bin/ctr/ctr_view.cgi?recptno=R000051635 (UMIN000045195; principal investigator: Atsuhiro Kitaura; registered on August 20, 2021). All patients who underwent transfemoral TAVR between April 2021 and December 2022 at Kindai University Hospital were retrospectively identified using the electronic medical records without an a priori sample size calculation. Patients were excluded if they (1) deviated from the protocol, (2) already had a strong inflammatory response confirmed by a preoperative blood test, (3) were poorly documented or required additional postoperative procedures, or (4) declined to provide information for the present study. Therefore, data from 143 patients were ultimately included and extracted from electronic charts.

#### 2.2.1 Anesthesia protocol

Patients were not administered any medication before TAVR. Vital sign monitors (electrocardiography, pulse oximetry, a noninvasive blood pressure monitor, and bispectral index monitor [Aspect Medical Systems, Inc.]) were placed on patients in the operating room. An arterial line was inserted under local anesthesia, and anesthesia was induced and maintained by remimazolam (R group) or dexmedetomidine (D group).

#### 2.2.2 Remimazolam anesthesia

In the R group, anesthesia was induced at 12 mg/kg/h and maintained at 1 mg/kg/h after confirming that Modified Observer's Assessment of Alertness/Sedation score (MOAA/S score) was <3.

#### 2.2.3 Dexmedetomidine anesthesia

In the D group, a loading dose of 4 μg/kg/min was administered for 10 min and the dose was subsequently reduced to 0.7 μg/kg/min. To achieve deep sedation, 20 mg propofol was used while administering the loading dose of dexmedetomidine. Propofol was used during urethral dilation balloon insertion, highly invasive procedures, such as rapid ventricular pacing, and in the event of body movements that interfered with the surgical procedure. The timing and dose of propofol were selected by the anesthesiologist in charge.

In both groups, patients were continuously administered remifentanil (0.03 μg/kg/min), local anesthesia with 1% lidocaine, and acetaminophen (15 mg/kg) for pain during the placement of a central venous catheter, surgical invasion, and other procedures. All patients were administered dexamethasone to prevent postoperative nausea and vomiting.

### 2.3 Outcome measures

The primary endpoint was the presence or absence of the antioxidant effects of the anesthetics themselves using ESR.

Secondary endpoints included the results of pre‐ and postoperative blood samples (C‐reactive protein [CRP] level and white blood cell [WBC] count). Based on blood test results (primarily CRP levels and WBC counts), patients with a weak inflammatory response preoperatively as defined by CRP ≤ 1 mg/dL and a WBC count of 3000−10,000 were examined in the present study. Measurements were taken preoperatively and on postoperative days 1 and 3 (POD1 and POD3) for comparisons.

### 2.4 Statistical analysis

Statistical analyses were performed using GraphPad Prism 9.5.1 (GraphPad Software, LLC). Data obtained in the present study were expressed as the mean ± standard deviation (SD) and between group differences were assessed using the paired or unpaired Student's *t*‐test. A one‐way analysis of variance (ANOVA) or repeated measures ANOVA followed by the Bonferroni multiple comparisons test was used as indicated. Categorical variables were presented as frequency (percentage) and between group differences were examined using the chi‐square test. *p* < .05 were considered to indicate a significant difference, and all *p* Values were two‐sided.

## 3 RESULTS

### 3.1 Laboratory study

#### 3.1.1 Measurement of ROS‐scavenging activities of anesthetic drugs by ESR

**Figure 1:**
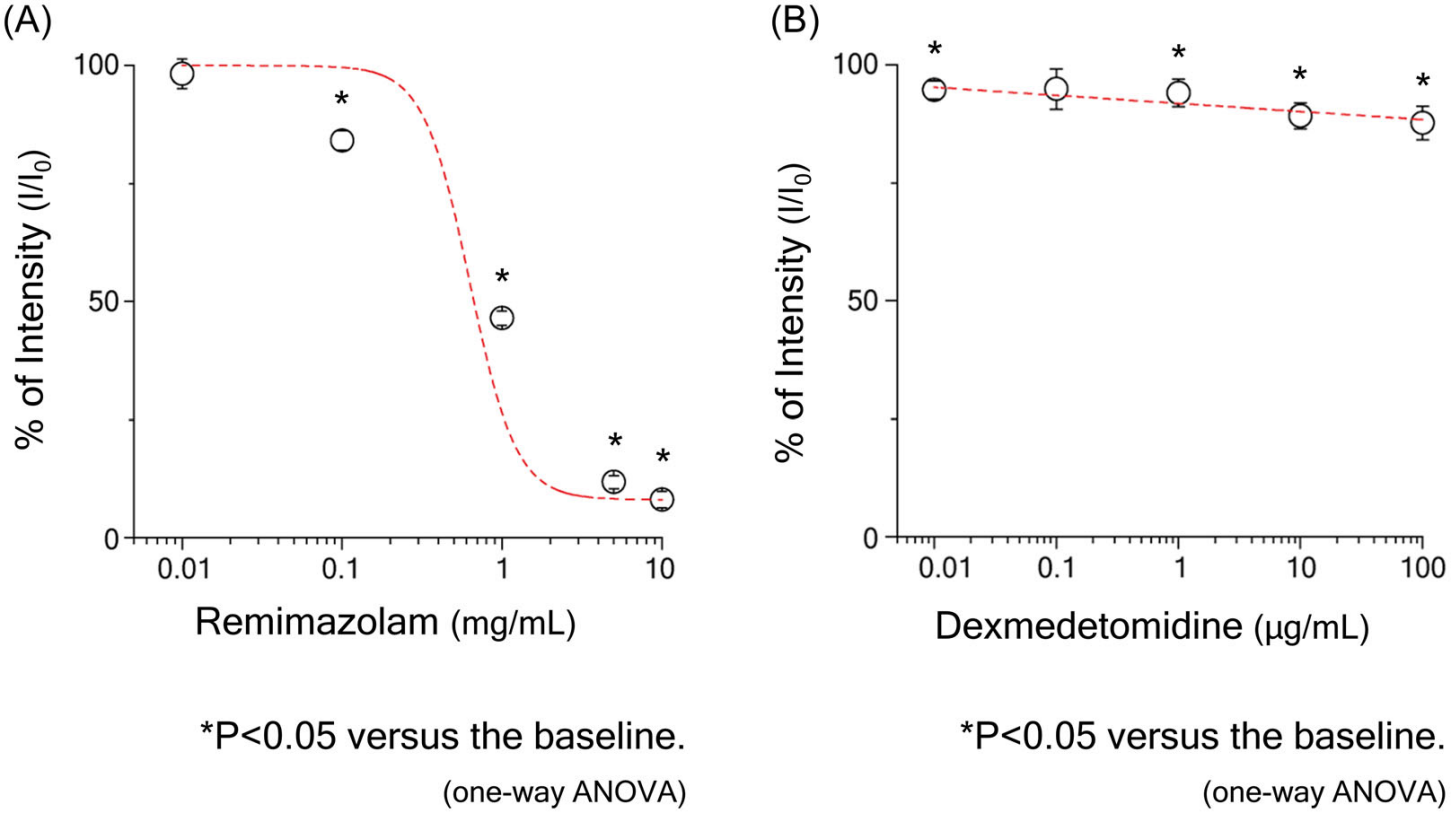
The spectral intensity of CYPMPO‐OH was examined under various concentrations of anesthetic drugs. It shows the results for remimazolam (Fig. 1A) and for dexmedetomidine (Fig.1B). To examine the relationship between intravenous anesthetic drug concentrations and spectral intensity, linear and nonlinear regressions were applied. The asterisk *indicates *p* < .05 and the double asterisk **indicates *p* < .01 versus the baseline (one‐way ANOVA). ANOVA, analysis of variance.

In Figure 1, the vertical axis represents the percentage of intensity (I/I_0_) and the horizontal axis shows anesthetic concentrations in log scale. Both remimazolam (Anerem®) and dexmedetomidine (Precedex®) scavenged OH^•^ in a concentration‐dependent manner. However, remimazolam at clinical concentrations was more capable of scavenging OH^•^ than dexmedetomidine.

### 3.2 Clinical study

#### 3.2.1 Patient demographics

**Figure 2:**
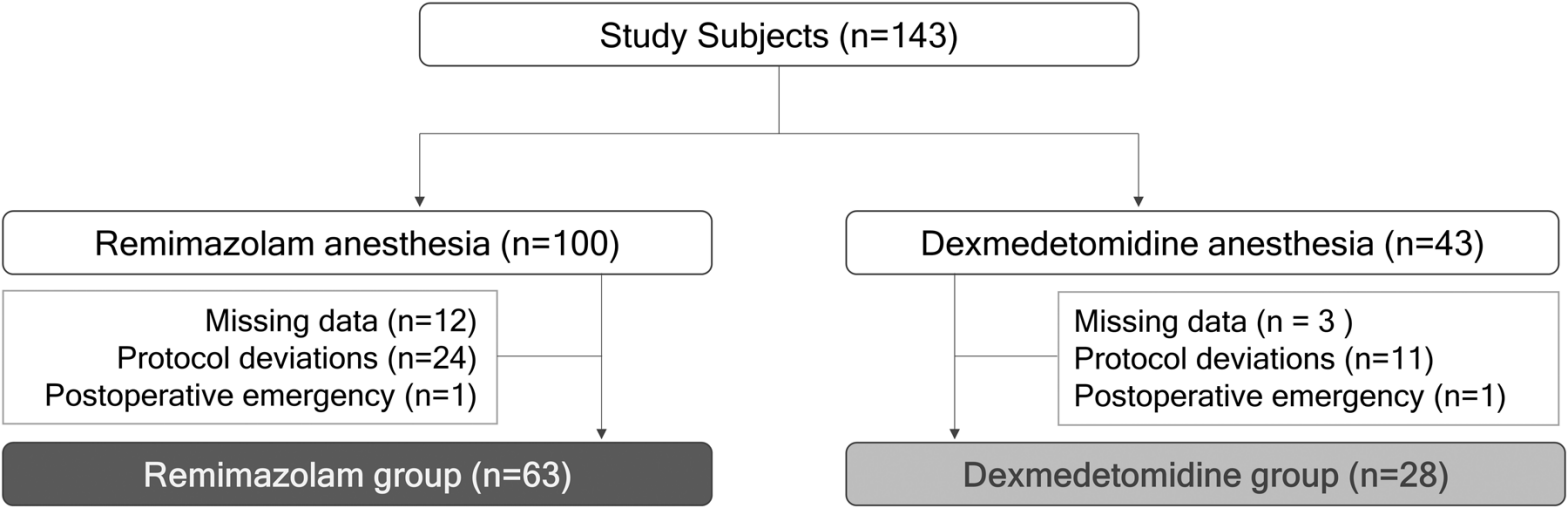
Flow chart of the present study.

Patients were classified into the R and D groups (63 and 28 patients respectively) according to the agent used during the induction and maintenance of anesthesia (Figure 2). No significant differences were observed in age, sex, BMI, the duration of operation, or the duration of anesthesia between the R and D groups (Table 1). The remifentanil dose was significantly higher in the R group than in the D group (0.98 ± 0.46 vs. 0.74 ± 0.43, *p* = .026 (unpaired *t*‐test)).

**Table 1:** Patient backgrounds in each group. Each parameter is expressed as a mean ± SD (paired *t*‐test) or as a number and % (chi‐square test).

	Remimazolam group (*n* = 63)	Dexmedetomidine group (*n* = 28)
Age	83.7 ± 5.9	84.1 ± 4.0
Sex (male/female)	26(41.3)/37(58.7)	9(32.1)/19(67.9)
BMI	22.4 ± 3.3	22.8 ± 3.1
Anesthesia duration (min.)	88.2 ± 20.0	88.8 ± 20.4
Operation duration (min.)	47.7 ± 16.4	49.7 ± 16.3
Remifentanil dose (mL)	0.98 ± 0.45	0.74 ± 0.43

*Note*: # indicates *p* < .05 versus the remimazolam group. No significant differences were observed between the two groups, except for the remifentanil dose.

#### 3.2.2 Inflammatory response during the perioperative period

**Figure 3:**
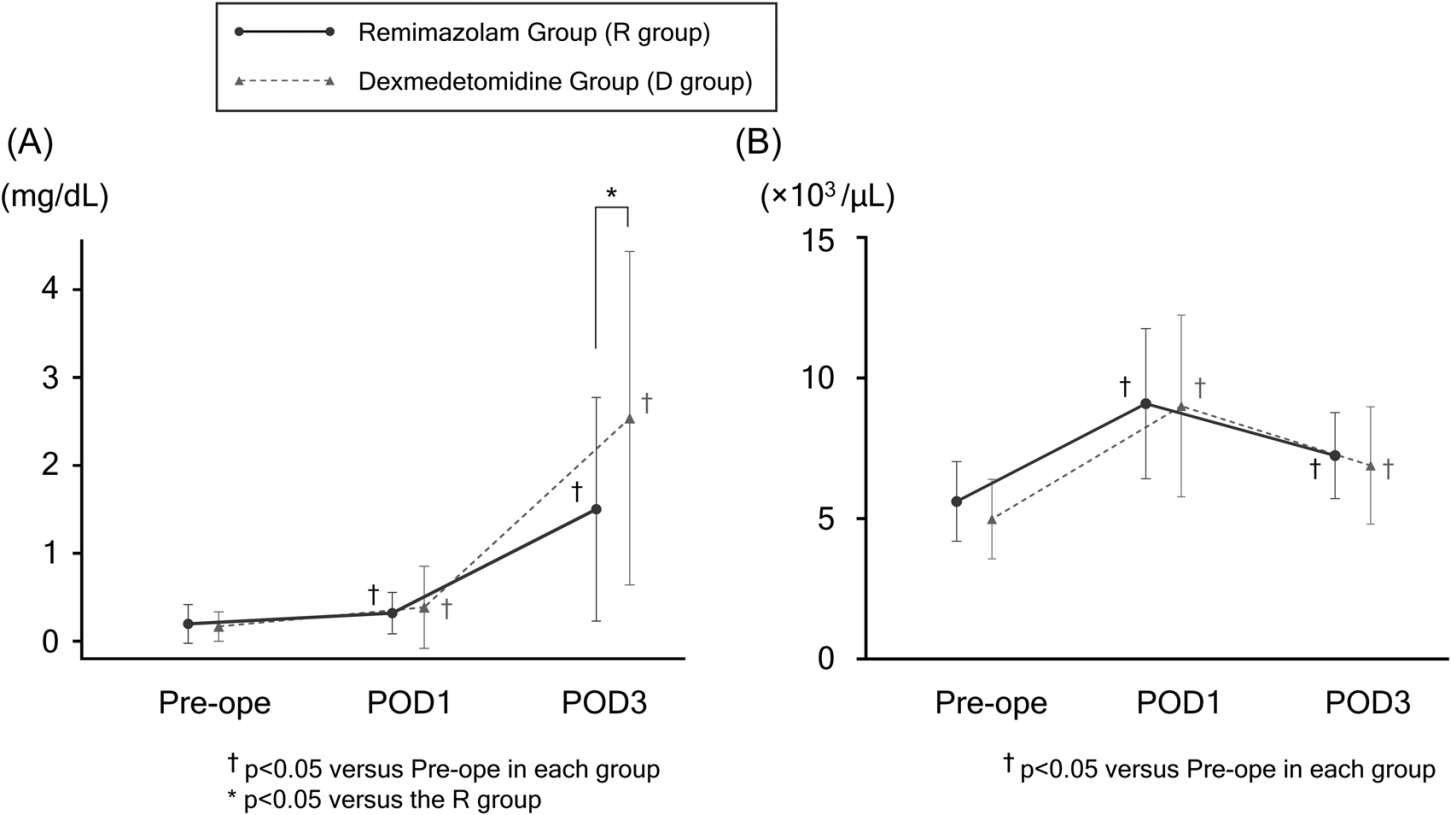
Changes in (A) the level of CRP and (B) WBC count from preoperatively (Pre‐ope) to postoperative days 1 and 3 (POD1 and POD3). In the graph, black circles and solid lines indicate the R group, and gray triangles and dotted lines indicate the D group. The asterisk *indicates *p* < .05 versus the R group (unpaired *t*‐test). The dagger (†) indicates *p* < .05 and versus Pre‐ope in each group (one‐way ANOVA). ANOVA, analysis of variance.

In both groups, a comparison with Pre‐Ope CRP levels (R group 0.18 ± 0.23, D group 0.17 ± 0.19) showed that CRP levels significantly increased on POD 1 (R group 0.49 ± 0.82, *p* = .01, D group 0.36 ± 0.42, *p* = .038) and POD3 (R group 1.33 ± 1.29, *p* < .01, D group 2.17 ± 1.84, *p* < .01). A significant difference in CRP levels was observed on POD3 between the R group (1.33 ± 1.29) and D group (2.17 ± 1.84, *p* = .014), but not Pre‐ope (0.18 ± 0.23 vs. 0.17 ± 0.19, *p* = .836) or on POD1 (0.49 ± 0.82 vs. 0.36 ± 0.42, *p* = .439) (Figure 3A).

Moreover, a comparison with Pre‐ope WBC counts in both groups (R group 5.7 ± 1.4, D group 5.2 ± 1.5) showed that WBC counts significantly increased on POD 1 (R group 9.0 ± 3.3, *p* < .001, D group 9.0 ± 3.1, *p* < .001) and decreased on POD3 (R group 7.3 ± 1.7, *p* < .001, D group 6.8 ± 2.4, *p* < .001). No significant differences were observed between the R and D groups Pre‐ope (5.7 ± 1.4 vs. 5.2 ± 1.5, *p* = .114) or on POD1 (9.0 ± 3.3 vs. 9.0 ± 3.1, *p* = .966) or POD3 (7.3 ± 1.7 vs. 6.8 ± 2.4, *p* = .349) (Figure 3B).

## 4 DISCUSSION

The present study investigated whether the antioxidant properties of anesthetics were involved in inhibiting the postoperative inflammatory response, and the following results were obtained:

(1) Remimazolam at clinical concentrations significantly scavenged OH•.
(2) On POD3, CRP levels were lower in the remimazolam group than in the dexmedetomidine group.
(3) CRP production may be suppressed by scavenging OH•.
(4) The anti‐inflammatory effects of dexmedetomidine were not associated with its antioxidant properties.

Invasive surgical procedures and the resulting inflammatory response activate macrophages locally, which leads to the production of humoral factors. These factors subsequently activate the endocrine, immune, and central nervous systems to elicit various biological responses.^22^, ^23^, ^24^ IL‐6 is an inflammatory cytokine that acts on the liver as an inflammatory response to surgical procedures and suppresses the synthesis of CRP and other proteins during the acute phase.^24^, ^25^, ^26^ Various cytokines induce complex signaling pathways and elicit a systemic inflammatory response, which may result in tissue damage and progression to multiple organ failure in some cases.^23^, ^26^ Previous studies demonstrated that anesthesia, invasive surgical procedures, and the use of sedatives in the intensive care unit may induce immunosuppression and lymphocyte dysfunction.^27^ Dexmedetomidine exerts anti‐inflammatory effects through changes in the production of inflammatory cytokines by macrophages and monocytes via the alpha‐2 adrenergic receptor.^15^, ^28^ Midazolam, a benzodiazepine drug similar to remimazolam, acts on central benzodiazepine receptors to exert sedative effects. Furthermore, it binds to peripheral benzodiazepine receptors and may exert anti‐inflammatory effects.^17^ Peripheral benzodiazepine receptors are also known as the 18‐kDa transporter protein (TSPO) and are present on liver macrophages.^18^ Midazolam suppresses NF‐κB, a gene that encodes inflammatory mediators, and inhibits macrophage‐mediated immune responses to elicit anti‐inflammatory effects through the activation of TSPO‐mediated signaling pathways.^17^, ^29^ A previous study demonstrated that remimazolam suppressed the phosphorylated p38 pathway by activating peripheral benzodiazepine receptors on hepatic macrophages at the cellular level, thereby reducing the levels of inflammatory cytokines produced.^18^ Based on these findings, both drugs appear to contribute to the receptor‐mediated suppression of acute inflammatory cytokine production and exert anti‐inflammatory effects.

Regarding anti‐inflammatory effects, we herein focused on the antioxidant properties of the anesthetic drugs themselves. A close relationship has been reported between oxidant stress and inflammation.^19^ OH^•^ is a ROS (antioxidants) that plays a critical role in inflammation. Phagocytes, such as neutrophils and macrophages, produce superoxide via NADPH oxidase in the cell membrane. H_2_O_2_ produced from superoxide generates OH^•^ by the Fenton reaction.^20^ In the liver, oxidative stress, including OH^•^, affects the production of inflammatory cytokines via the NF‐kB signaling pathway, which, in turn, inhibits the production of CRP.^30^ The present results indicate that remimazolam (Anerem®) at clinical concentrations inhibited the activation of the NF‐kB pathway in hepatocytes by scavenging OH^•^ produced in blood vessels. In contrast, dexmedetomidine (Precedex®) did not scavenge OH^•^ at clinical concentrations. The different effects of these drugs may have resulted in differences in CRP production.

The present results also demonstrated that the dose of remifentanil was significantly higher in the R group. Previous studies reported that remifentanil did not affect the production of CRP.^31^, ^32^ Therefore, the difference in the amount of remifentanil used between the R and D groups may not have been a contributing factor to the reduction observed in CRP levels. However, the inhibitory effects of remimazolam on body movements may have been weaker than those of propofol,^2^ which may have necessitated a higher dose of remifentanil.

Blood samples showed that remimazolam suppressed CRP production on POD3. In a previous study on coronary artery bypass surgery, postoperative CRP peaked on POD2.^32^, ^33^ After 1 week of observation following laparoscopic colorectal surgery, the highest postoperative CRP level was detected on POD2.^34^ However, in this retrospective study, blood samples were only available on POD3; therefore, data obtained on POD1 and POD3 were analyzed.

There are a number of limitations that need to be addressed. This was a retrospective analysis and the inflammatory response was assessed based solely on CRP levels and WBC counts. Furthermore, blood samples showed that remimazolam suppressed CRP production on POD3. In a previous study on coronary artery bypass surgery, postoperative CRP levels peaked on POD2. Furthermore, another study measured CRP levels for 1 week after laparoscopic colorectal surgery, and detected the highest levels on POD2. However, in the present study, blood samples were only available on POD1 and POD3. Another limitation is that the anesthetic drugs used for ESR were commercialized. Anerem® comprises lactose hydrate, dextran, and a pH adjuster, while Precedex® contains sodium chloride. These additives may have exerted effects that cannot be ruled out. Moreover, dexmedetomidine (Precedex®) is in a liquid form that has a maximum concentration of approximately 0.5 mmol/L. In the present study, experiments were conducted at the maximum concentration for clinical use. Concentrations higher than those used in clinical settings may exert antioxidant effects.

## 5 CONCLUSIONS

Remimazolam (Anerem®) was shown to inhibit CRP production to the same extent as dexmedetomidine (Precedex®), which is already known to exhibit anti‐inflammatory activity. This anti‐inflammatory activity may be attributed to an enhanced antioxidant effect.

Since dexmedetomidine at clinical concentrations does not exert antioxidant effects, its anti‐inflammatory activity is independent of these effects.

## AUTHOR CONTRIBUTIONS

Shota Tsukimoto, Atsuhiro Kitaura, and Takuro Sanuki conceived and designed the study. In the laboratory study, Shota Tsukimoto, Hidetaka Kuroda, and Ayaka Yoshida prepared materials and collected data, Shota Tsukimoto and Hidetaka Kuroda performed experiments and analyzed data, and Ayaka Yoshida and Fumihiko Yoshino supervised the study. In the clinical study, Atsuhiro Kitaura performed experiments and collected clinical data, Shota Tsukimoto analyzed data, and Shinchi Nakao, Noriyuki Ohta, and Yasuhumi Nakajima supervised the study. Shota Tsukimoto wrote the first draft of the manuscript, which was edited by Uno Imaizumi and Takuro Sanuki. The manuscript was reviewed and revised by all co‐authors. The final version of the manuscript was read and approved by all authors.

## CONFLICT OF INTEREST STATEMENT

The authors declare no conflict of interest.

## ETHICS STATEMENT

The Research Ethics Board of Kindai University Faculty of Medicine approved the present study (approval no. R03‐123), which was performed according to the principles of the Declaration of Helsinki. we followed the opt‐out model with approval of IRB of Kindai University Faculty of Medicine (https://www.med.kindai.ac.jp/anes/clinical_study.html). The participant has consented to the submission of the article to the journal.

## Supporting information

Supporting information.

## Data Availability

The datasets generated during and/or analyzed during the current study are available from the corresponding author on reasonable request.
